# Understanding Aberrant Signaling to Elude Therapy Escape Mechanisms in Myeloproliferative Neoplasms

**DOI:** 10.3390/cancers14040972

**Published:** 2022-02-15

**Authors:** Maria Teresa Bochicchio, Valeria Di Battista, Pietro Poggio, Giovanna Carrà, Alessandro Morotti, Mara Brancaccio, Alessandro Lucchesi

**Affiliations:** 1 Biosciences Laboratory, IRCCS Istituto Scientifico Romagnolo per lo Studio dei Tumori (IRST) “Dino Amadori”, 47014 Meldola, Italy; teresa.bochicchio@irst.emr.it; 2 Hematology Unit, IRCCS Istituto Scientifico Romagnolo per lo Studio dei Tumori (IRST) “Dino Amadori”, 47014 Meldola, Italy; valeria.dibattista@irst.emr.it; 3 Department of Molecular Biotechnology and Health Sciences, University of Torino, 10126 Torino, Italy; pietro.poggio@unito.it; 4 Department of Clinical and Biological Sciences, University of Torino, 10043 Orbassano, Italy; giovanna.carra@unito.it

**Keywords:** myeloproliferative neoplasms, JAK2, Aurora A, ROCK, drug resistance, cell signaling

## Abstract

**Simple Summary:**

Myeloproliferative neoplasms are a group of rare disorders characterized by genetic mutations in hematopoietic stem cells and by the presence of systemic inflammation. The main driver mutations causing these diseases converge in activating the JAK2 signal transduction pathway, which plays a major role in disease onset and maintenance. Treatments based on JAK2 inhibitors ameliorate symptoms without suppressing the disease. This depends on the reactivation of JAK2 signaling and on the emergence of alternative pathways also sustained by inflammatory mediators. Molecular mechanisms at the basis of disease persistence and new therapeutic attempts to overcome them are discussed in the review.

**Abstract:**

Aberrant signaling in myeloproliferative neoplasms may arise from alterations in genes coding for signal transduction proteins or epigenetic regulators. Both mutated and normal cells cooperate, altering fragile balances in bone marrow niches and fueling persistent inflammation through paracrine or systemic signals. Despite the hopes placed in targeted therapies, myeloid proliferative neoplasms remain incurable diseases in patients not eligible for stem cell transplantation. Due to the emergence of drug resistance, patient management is often very difficult in the long term. Unexpected connections among signal transduction pathways highlighted in neoplastic cells suggest new strategies to overcome neoplastic cell adaptation.

## 1. Introduction

Myeloproliferative neoplasms (MPNs) are clonal stem cell diseases characterized by distinct hematological and clinicopathologic features. On the base of the predominant terminally differentiated myeloid cell involved in the malignancy, MPNs are classified in different subtypes: polycythemia vera (PV), essential thrombocythemia (ET), and primary myelofibrosis (PMF). Since 2005, starting with the description of the Janus kinase 2 mutation (JAK2V617F) [1,2] followed by the discovery of myeloproliferative leukemia virus oncogene (*MPL*) [3] and calreticulin (*CALR*) mutations [4,5], the mutational landscape of MPNs has expanded enormously. Indeed, mutations in the so-called “high molecular risk” genes (HMR) (*IDH1/2*, *EZH2*, *ASXL1*, and *SRSF2*) have been associated with disease progression, and should be sought in PMF patients who are transplant candidates [6]. Particular attention should also be paid to the detection of DTA mutations (DNMT3A, TET2, and ASXL1) associated with clonal myelopoiesis, which sometimes precede the appearance of driver mutations, a more aggressive clinical course, and “overlap” syndromes with dysplastic aspects [7]. These genetic discoveries and the correlation studies between the genotype and phenotype have modified clinical practice, providing new diagnostic and prognostic opportunities.

However, we have to overcome a totally mechanistic paradigm, which is based solely on the serial accumulation of point mutations, by recognizing dysimmunity and inflammation as the other key elements of the natural history of MPNs. As evidence of this, the most important clinical implications are the promotion of atherosclerosis, thrombosis, and symptoms due to an excessive release of proinflammatory cytokines. All MPN patients, mainly PV patients, are, in fact, at an increased risk of developing thrombotic or hemorrhagic complications compared to the general population [8]. PV and ET can evolve to myelofibrosis (MF), and all these MPNs can eventually progress into acute myeloid leukemia (AML), which is usually refractory to intensive chemotherapy [9]. PMF patients suffer from constitutional symptoms such as fatigue, night sweats, fevers, weight loss, pruritus, and bone pain. Moreover, symptomatic hepatosplenomegaly and cytopenia also negatively affect their quality of life. The normalization of blood counts and alleviation of MPN-related symptoms including constitutional symptoms, spleen-related symptoms, and the prevention of thrombosis and disease progression represent, therefore, the mainstay of MPN treatment. Available treatment options can include phlebotomy, and myelosuppressive therapies such as hydroxyurea, oral busulfan, anagrelide, interferon, and the JAK1/2 inhibitors ruxolitinib and fedratinib. Erythropoiesis-stimulating agents (ESAs), androgens, prednisone and immune modulatory agents such as thalidomide and lenalidomide are reserved for MF patients with anemia and thrombocytopenia. The only curative treatment for the MPNs is allogeneic stem cell transplantation (ASCT), even if it is restricted only to a limited number of young patients, in the absence of comorbidity, and with a suitable donor. The choice of therapy is based on a variety of stratification tools that offer prognostic scores capable of assessing the risk of blastic transformation, evolution in MF or thrombosis development [10,11,12,13,14,15]. The parameters considered for risk calculation include age, clinical characteristics, cytogenetic abnormalities and the presence of HMR mutations. In 2009, the European LeukemiaNet (ELN) Consortium proposed criteria for response assessment in both ET and PV [16]. While effective in the attempt to provide clues for response, these criteria were unable to directly measure clinical outcomes. In particular, thrombosis risks and survival were not evaluated. More recently, a revised recommendation has been published [17]. These novel criteria include both the assessment of antiproliferative response and the long-term effects of the drugs. Specifically, these investigations measured the normalization of symptoms and signs of the disease, remission of peripheral blood counts, absence of vascular events without signs of disease progression, and bone marrow histological abnormalities. Notably, such criteria are different in PV and in ET.

Despite the huge progress in understanding the pathophysiology of MPNs that has led to an increase of the available therapies, to date none of them have been demonstrated to arrest the progression of these diseases except for a transient improvement of the systemic symptoms and of the cytopenia and a reduction of splenomegaly. The possible explanation lies in the biological complexity, which originates from the mutational profile and ultimately affects it, but which is fueled by the peculiar immunological imbalances and inflammation. These elements recall what is observed in rheumatic diseases, in which autoimmunity represents itself a valid model of carcinogenesis. The activation of the inflammasome in MPNs insists on a situation of pre-existing genetic instability, with foreseeable risks and consequences [18,19].

The research of new drugs capable of overcoming the resistance to these treatments, modifying the disease course, represents an area of intense investigation. The most promising way forward, given the current developments in the clinical trial landscape, could be combination therapies. In this, JAK inhibitors could represent the foundation stone on which to build new multi-kinase inhibition strategies.

## 2. Mutational Landscape at a Glance

Driver mutations causing MPNs include *JAK2* V617F and exon 12 mutations, *MPL* gene mutation in exon 10 and frameshift mutations in exon 9 of the *CALR* gene. All these mutations converge in activating the JAK2/STAT pathway and cause myeloid cell hyperproliferation [20]. In addition, MPN patients could be characterized by the presence of other alterations that are able to enhance and sustain driver mutation signaling. These additional mutations involve epigenetic regulators, signaling pathways and splicing machinery [21].

*ASXL1*, *EZH2* and *IDH1/2* gene mutations are associated with poor outcomes and a shorter time to treatment discontinuation (TTD) and overall survival (OS) in MF patients [22]. Although RAS pathway mutations affect only 1% of PV and ET and about 5% of MF [23], with a positive association between RAS pathway and HMR mutations, an elevated white blood cell count (WBC) and progression to AML have been reported [24]. Moreover, AKT and extracellular signal-regulated kinase (ERK) activation and RAS-activating mutations have been demonstrated to be responsible for JAK inhibitor ruxolitinib (INCB018424) resistance by the inhibition of the phosphorylation of BAD [25].

Splicing factors represent the second most frequently mutated genes in MPNs, suggesting that abnormal splicing plays a key role in the evolution of chronic myeloproliferative diseases [26,27]. PV and PMF patients with *SRSF2* mutations have inferior overall survival and leukemia-free survival rates [28]. Moreover, high-throughput next-generation sequencing (NGS) revealed that about 20% of MPN patients harboring *SRSF2* mutations evolved into acute leukemia [29].

Besides somatic mutations, germline mutations in *RBBP6* and *GSKIP* genes have been investigated. They disrupt the response to the apoptotic stimuli, impairing the p53 pathway and increasing the risk of developing further mutations and thrombopoietin sensitivity mediated by *ATG2B* and *GSKIP* overexpression [30].

## 3. Resistance to JAK Inhibitors

The comprehension of the molecular drivers responsible for the onset of MPNs represents an important step forward in the generation of targeted therapies. The presence of mutations in genes coding for MPL, JAK2 and CALR clearly point to a main involvement of the JAK2 pathway. In vitro and in vivo experiments further sustain this view, demonstrating that these mutations are sufficient to drive the pathology [31,32]. The fact that cells carrying these mutations become addicted to JAK2 pathway hyperactivation provided the rationale for generating JAK2 inhibitors [33] (Table 1). Ruxolitinib and fedratinib are JAK2 inhibitors approved for clinical use in MPNs. Despite their ability to reduce splenomegaly and general symptoms, their limited efficacy in eradicating mutant clones and their adaptation to JAK2 inhibition make the pathology still incurable. Under the selective pressure of tyrosine kinase inhibitors (TKIs), cells could adapt to survive and to proliferate even when JAK2 signaling is inhibited [34,35].

### 3.1. Genetic Mechanisms of Resistance

The oncogenic properties of JAK2 belong mostly to its tyrosine kinase activity. As observed with various tyrosine kinases, prolonged treatment with JAK2 inhibitors allow the emergence of acquired second-site mutations, with reduced sensitivity to the inhibitors. Various mutations have been described in *JAK2* as being able to confer resistance to JAK2 inhibitors. A number of in vitro randomly generated *JAK2* mutations, including Y931C, G935R, R938L, E864K, I960V, and E985K, sustain cellular growth by the phosphorylation of *JAK2* downstream substrates also in presence of the inhibitor [47,48]. Almost all the identified mutations are in the kinase domain of *JAK2* and are relatively few when compared to *BCR-ABL1* mutations [47,49,50]. Other *JAK2* resistance-related mutations, such as G993A or L884P, have also been reported in cell models of acute lymphoblastic leukemia (ALL) [51]. Rarely, uncommon *JAK2* germline mutations cause hereditary thrombocytosis resistant to JAK2 inhibitors [49].

In addition, it is worth noting that besides *JAK2*, various other genes have been found mutated [52] and the order of mutation presentations has been postulated to be of clinical relevance [53]. It is therefore tempting to assume that JAK2 inhibitor resistance or, more generally, JAK2 inhibitor responses, could simply be a consequence of the genetic landscape of individual MPN clones.

### 3.2. JAK2 Signaling

**Figure 1:**
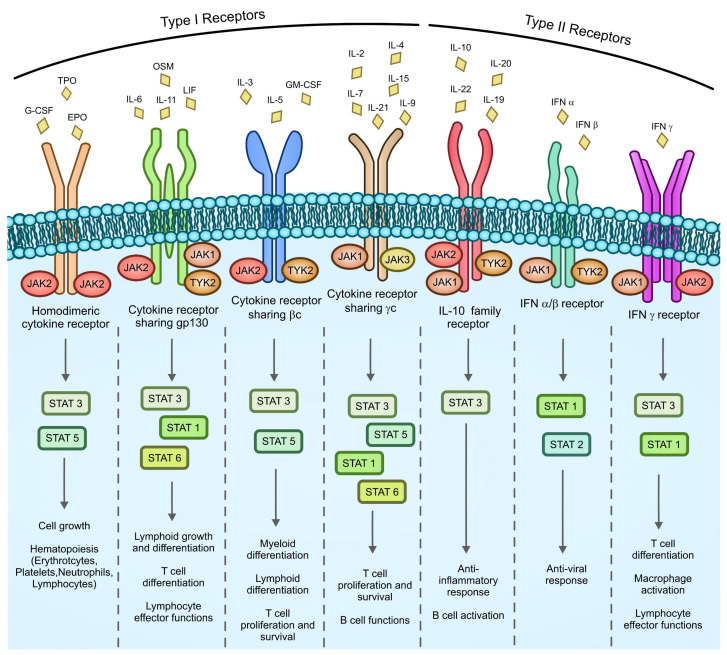
Cytokine receptor signaling in hematopoietic cells. More than 50 different cytokines are coded by the human genome. Cytokine receptors are particularly important in maintaining hematopoietic cell physiological functions. They are divided into two subfamilies (type I and type II receptors) based on the structure of the extracellular domain. In response to cytokine recognition, each receptor can bind different combinations of JAKs that, in turn, besides activating the MAPK and PI3K/AKT pathways (see Figure 2), may phosphorylate different STATs. EPO, erythropoietin; G-CSF, granulocyte colony-stimulating factor; GM-CSF, granulocyte–macrophage colony-stimulating factor; IFN, interferon; LIF, leukemia inhibitory factor; OSM, oncostatin M; TPO, thrombopoietin.

JAKs are activated by type I and type II cytokine receptors. These receptors are composed by two or more protein chains containing a region termed the cytokine receptor homology region (CHR) in their extracellular domains. The junction in between the two CHRs forms the cytokine binding site. CHRs of type I cytokine receptors contain a conserved aminoacidic sequence called the “WSXWS motif”, which is not present in type II receptors. Both type I and type II receptors contain sequences in their cytoplasmic region able to bind JAKs and to recruit STATs [54,55]. Type I cytokine receptors can be formed by four major protein chains: alpha, beta common (βc), gamma common (γc) and gp130. These chains can form homodimeric receptors (consisting of two identical chains) or non-homodimeric receptors where a cytokine-specific chain (usually alpha) recruits a “shared” chain to initiate signaling. Homodimeric cytokine receptors can recognize different cytokines, such as EPO, TPO, and G-CSF. Receptors sharing the common gp130 chain recognize the IL-6 family of cytokines (IL-6, IL-11, LIF, and OSM) while the IL-3 family (IL-3, IL-5, and GM-CSF) signals via receptors that contain the βc chain. Finally, the IL-2 family of cytokines (IL-2, IL-4, IL-7, IL-9, IL-15, and IL-21) is recognized by receptors that contain the γc chain, a JAK3-associated receptor subunit. Similar to type I, the type II receptor family consists of both shared chains and cytokine-specific chains. Type II cytokines receptors are able to recognize interferons (IFN) and the IL-10 family of cytokines [54]. JAK kinases (JAK1, JAK2, JAK3, and TYK2) can directly bind to the intracellular domain of type I and II cytokine receptors. Different JAKs can associate with different receptors and phosphorylate distinct STAT family members (STAT1, 2, 3, 4, 5a, 5b, and 6) (Figure 1) giving specificity to the signals activated by cytokines [56,57,58]. Once activated, STATs dimerize and translocate into the nucleus where they modulate the gene transcriptions of a repertoire of target genes that can vary, for a given STAT, from one cell type to another [59].

**Figure 2:**
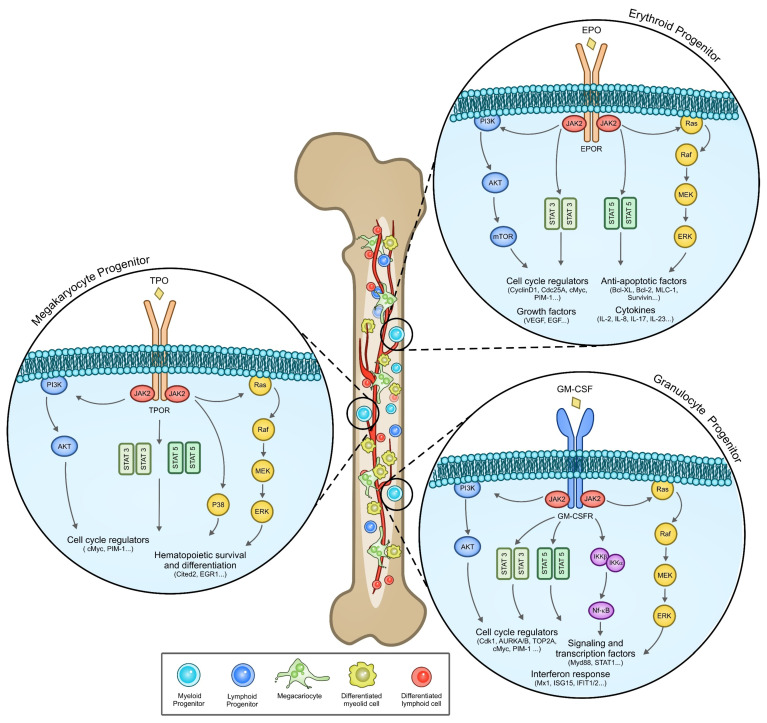
JAK2 signaling activated by three different surface receptors (EPR, TPO, and GM-CSF). The figure shows the signal transduction pathways induced by JAK2 signaling activated by three different receptors. Gene targets often code for proteins involved in cell differentiation, survival and proliferation. The overexpression of these genes in the pathological context contributes to the deregulation of homeostasis in the hematopoietic compartment and to the onset of the pathology [60,61,62,63,64,65].

In physiological conditions, JAK2 proteins, which are stably bound to the cytoplasmic region of several type I and II receptors [54], get activated in response to ligand binding and promote their own phosphorylation and the phosphorylation of tyrosine residues in the cytoplasmic region of the receptor. STATs bind through their SH2 domains to these phosphorylated tyrosines and get phosphorylated by JAKs. Phosphorylated STATs detach from the receptor, dimerize, translocate into the nucleus and activate the transcriptions of target genes (Figure 2).

In addition, JAK2 activates the MAPK pathway, leading to the phosphorylation of ERK, and the phosphatidylinositol 3-kinase (PI3K) pathway, promoting the activation of AKT and of the mammalian target of rapamycin (mTOR) [62] (Figure 2).

JAK2 has even been found to localize inside the nucleus of hematopoietic cells [66], opening a debate in the scientific community [67,68]. In response to IL-6, nuclear JAK2 has been found to phosphorylate Lysine Demethylase 3A (KDM3A). Phosphorylated KDM3A binds to STAT3 and is recruited to the promoters of the STAT3 target genes, where it decreases histone H3K9 methylation. Overall, KDM3A seems to function as a co-activator in mediating STAT3 target gene transcription [69]. A further nuclear substrate of JAK2 is PRMT5, a type II arginine methyltransferase able to methylate H2A, H3, and H4 [70], splicing factors and ribosome components [71,72].

The pathological overactivation of JAK2 represents a key event in the onset, maintenance and persistence of MPNs. The majority of JAK2-activating mutations fall in the pseudokinase domain, an autoinhibitory domain known to inactivate JAK2 kinase activity when cytokines bind to the receptor [73,74]. Recently, it has been demonstrated that cytokine receptor transmembrane domains and JAK2 pseudosubstrate domains cooperate in mediating receptor dimerization in response to cytokine binding [75]. Activating mutations in the JAK2 pseudokinase domain stabilize intermolecular interactions between JAK2, leading to constitutive receptor dimerization [75] and persistent downstream signaling.

**Figure 3:**
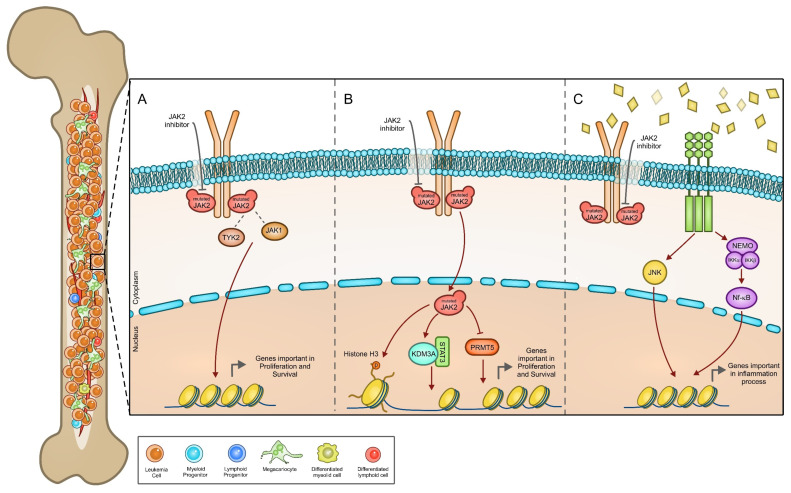
Altered molecular signaling pathways in resistant MPNs: (**A**) Mutant JAK2 can be activated through the formation of heterodimers with JAK1 or TYK2, thus sustaining STAT activation in the presence of JAK2 inhibitors. (**B**) JAK2 mutation can promote its translocation into the nucleus, where it phosphorylates different substrates (Histone H3, KDM3A, and PRMT5), modifying the epigenetic landscape of hematopoietic cells, thus promoting cancer cell proliferation and survival even in the presence of JAK2 inhibitors. (**C**) Besides JAK2 activation, aberrant cytokine release in MPNs triggers different intracellular pathways (JNK, NF-κB) able to sustain MPN malignant progression.

Rinaldi et al. demonstrated that mutated JAK2 is mainly nuclear in CD34^+^ cells derived from MPN patients, but not in differentiated cells [76]. When in the nucleus, mutant JAK2 has been described to acquire different functions (Figure 3B). JAK2 phosphorylates histone H3, impairing the binding of the heterochromatin protein 1 α (HP1 α) to the histone. HP1α is required for DNA packaging and gene silencing; thus, its failure to bind to the chromatin promotes the expression of the *lmo2* gene [66]. The gene, *lmo2*, is required for normal hematopoiesis [77] and exerts an oncogenic function in leukemia [78].

Mutant JAK2 binds with higher affinity to PRMT5 in respect to wild-type JAK2 and it inhibits PRMT5 methyltransferase functions through its phosphorylation [79]. Overall, the mutant JAK2, besides activating the STAT, MAPK and PI3K pathways, may influence global gene transcription by altering histone methylation and controlling the activity of other nuclear substrates, causing defects in splicing fidelity, protein translation, and growth factor signaling [71].

As observed in chronic myeloid leukemia (CML) [80], various functional mechanisms have also been postulated to overcome sensitivity to JAK2 inhibitors, representing the most clinically relevant causes of resistance. The reactivation of the JAK2/STAT pathway and the concomitant activation of alternative pathways have been highlighted in response to prolonged treatment with ruxolitinib. Indeed, the compensatory stimulation of the MAPK pathway clearly reduces the efficacy of JAK2 inhibitors both in vitro and in MPN mouse models [81,82]. The use of inhibitors of these additional pathways attracted interest in the field [83,84] that is now translating into the clinic (Table 2). For this reason, dissecting the mechanisms at the basis of MAPK signaling persistence in JAK2-mutated cells is of particular significance. Using a phosphoproteomic approach, Jayavelu et al. identified several proteins involved in mRNA processing as a target of mutated JAK2. Among them, the splicing factor Y-box-binding protein 1 (YBX1) has been proved to play a crucial role in disease persistence [71]. YBX1 phosphorylation depends on JAK2 and MAPK-interacting serine/threonine kinase 1 (MKNK1) activity. YBX1 is required for ERK activation in JAK2-mutated cells and for ERK signaling maintenance during treatment with JAK2 inhibitors. Of note, YBX1 inactivation sensitizes JAK2 V617F to JAK inhibitors by inducing cell death [71]. The main JAK2 downstream targets involved in disease persistence are listed in Table 3.

Paradoxically, mutant JAK2 has been shown to be overexpressed and hyperphosphorylated in cells treated with ruxolitinib and other JAK2 type I inhibitors. These types of inhibitors are ATP-competitor compounds, able to stabilize the kinase in its active conformation and block its downstream signaling [90,91]. The increased phosphorylation of the JAK2 activation loop seems to be dependent on the binding mode of the type I inhibitor; it is not mediated by type II inhibitors, which stabilize JAK2 in its inactive state [91]. It has been demonstrated that the reactivation of the JAK/STAT pathway after prolonged ruxolitinib treatment depends on the formation of heterodimers between JAK2 and TYK2 or JAK1 and their subsequent transphosphorylation [35,90] (Figure 3A). However, mutant cells retain their dependence on JAK2 activation, being sensitive to JAK2 downregulation or degradation [90]. Ruxolitinib treatment also results in increased JAK2 transcription and post-translational stabilization [90]. Indeed, Tvorogov et al. demonstrated that ruxolitinib binding to JAK2 impairs JAK2 dephosphorylation and blocks its ubiquitination and degradation [92]. In this regard, the possibility to induce JAK2 degradation by means of HSP90 inhibitors [90], or by proteolysis-targeting chimera (PROTAC) technology [93,94,95], is an interesting perspective for the development of innovative treatments.

The ability of active JAK2 to regulate chromatin accessibility and affect global gene expression is of crucial interest to understand MPN pathophysiology. These alterations may also account for the development of mechanisms of adaptation to treatment with JAK2 inhibitors and for the onset of resistance. Indeed, treatment with ruxolitinib causes alterations in histone methylations in MPN cell lines and patients’ blood and bone marrow cells [96]. Other genes coding for proteins involved in histone or DNA methylation have been found recurrently mutated in MPN patients, such as *ASXL1*, *TET2*, *DNMT3A*, and *EZH2* [97,98,99,100], raising the complexity of the epigenetic alterations in MPN patients. These mutations are not restricted to MPNs and have been found in several myeloproliferative disorders. Of note, they may occur before or after *JAK2* and *CALR* mutations or may co-exist in separate clones [101,102]. Indeed, these mutations together with others (*ASXL1, SRSF2, CBL, KIT, RUNX1, SH2B3*, and *CEBPA*) impact on the evolution of the disease, and the timing of occurrence influences the disease phenotype [62,102,103,104,105].

The fact that both the overactivation of the JAK2 pathway and the additional mutations arising in MPN patients affect epigenetic regulation strongly suggests that epigenetic aberrations play a relevant part in MPN pathophysiology and in the onset of resistance to therapies. The combination of JAK inhibitors with epigenetic therapies is under investigation and appears to yield interesting results [102].

### 3.3. Cytokine Deregulation

The disturbance of the immunological system and the inappropriate release of cytokines may alter the complex equilibrium among progenitor cell proliferation, the differentiation toward specific lineages and cell death. Indeed, increasing evidence indicates that the aberrant production of cytokines is crucial for the development and persistence of MPNs [106]. These cytokines may act on neoplastic cells or through paracrine signals on normal hematopoietic cells and bone marrow stromal cells. Further, cytokine deregulation in systemic circulation disturbs the physiological crosstalk among different organs.

The role of these inflammatory mediators in sustaining malignant clone survival and expansion or promoting specific pathological features has been assessed by different studies [106,107,108]. An aberrant release of cytokines, in the absence of articulated feedback programs aimed at promoting resolution, is the cause of a self-maintaining chronic inflammatory state in these patients. Cytokines, once released, may bind with high affinity to surface receptors expressed in several cell types, triggering signal transduction pathways, such as JAK/STAT, NF-κB, JUNK, and others, which in turn promote cytokine release and propagation on inflammatory signals (Figure 3C). This generates a self-feeding vicious cycle, promoting gene mutations [109] and altering microenvironments to sustain cell proliferation and survival. Indeed, chronic inflammation and history of autoimmune diseases have been correlated with an increased risk of MPN onset and vice versa [110]. Signs of immune reactivity in the bone marrow of patients affected by myelofibrosis support the possibility that autoimmunity may play a role [110]. It is to be noted that autoimmune myelofibrosis exists as a separate clinical entity characterized by the presence of autoantibodies, diffuse fibrosis and benign clinical course [111]. All these considerations indicate that cytokine overproduction may represent an important predisposing factor to MPNs. The first indication comes from the presence of an abnormal amount of cytokines in the blood and bone marrow of MPN patients and MPN preclinical models [112,113,114]. The fact that patients without mutations in *JAK2*, *MPL* and *CALR* (affected by the so called “triple-negative MPNs”) show lower cytokine levels, indicates that, at least partially, cytokine production depends on the overactivation of the JAK2 pathway [115]. *STAT3* gene ablation in hematopoietic cells decreases cytokine levels in MPN mouse models and ameliorates disease symptoms, while STAT3 deletion restricted to MPN mutant cells does not alter disease severity, suggesting that the contribution of normal hematopoietic cells is requested for the development of the disease [114]. However, cytokines remain abnormally elevated in the blood of MPN patients treated with ruxolitinib, suggesting that other signaling pathways may contribute to maintain aberrant cytokine production [116].

Cytokines themselves may activate the NF-κB pathway in neoplastic and normal cells. For instance, IL-1 and TNF-α are often significantly elevated in MPNs, activating the NF-κB pathway by binding cell surface receptors in a variety of cell types, and inducing CD34^+^ cell survival and JAK2 V617F clonal expansion in MPN patients [117,118,119]. Indeed, an overactivation of the NF-κB pathway has been described both in neoplastic and normal cells [120,121]. Interestingly, megakaryocyte–erythroid progenitor cells derived from MPL W515L-diseased mice showed a deregulation in epigenetic marks in the regulatory region associated with the TNF-α/NF-κB signaling pathway and a marked increase in gene expression from these loci [121]. Bromodomain and extra-terminal (BET) domain 4 (BRD4), by binding to acetylated RelA and increasing its activity, is required for NF-κB-driven gene transcription [122]. The use of BET inhibitors has been proved to attenuate NF-κB activation. Inhibition of NF-κB transcriptional activity using BET inhibitors showed therapeutic efficacy in reducing inflammation and spleen weight and prolonging mice survival. This suggests that NF-κB plays a crucial role in sustaining MPNs and may represent an important target together with JAK2. Indeed, the combination of JAK and BET inhibitors gave very promising results in MPN preclinical models, promoting a great reduction in cytokines and white blood cells, spleen normalization, bone marrow fibrosis, and disease burden [121]. Similarly, to the effect of JAK2 type I inhibitors on JAK2, BET inhibitors may induce BET stabilization and accumulation, limiting their efficacy [123,124]. The development of PROTAC-based strategies may represent a further improvement in NF-κB inhibition and cytokine reduction [124,125].

Transforming growth factor β (TGF-β) is a cytokine upregulated in the bone marrow of patients with myelofibrosis, known to inhibit normal hematopoiesis and to promote extracellular matrix synthesis and deposition [126]. In vivo experiments in MF mouse models demonstrated the crucial role of TGF-β in the development of the pathology [127] and the potential of therapeutic approaches targeting TGF-β in blocking bone marrow fibrosis [128,129].

### 3.4. Aurora A and ROCK

**Figure 4:**
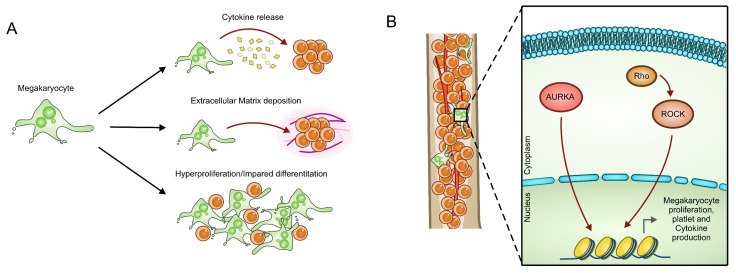
Megakaryocyte role in MPN progression and drug resistance. (**A**) Megakaryocytes play different roles in sustaining MPN cell malignancy. Abnormal megakaryocyte differentiation and functionality are considered responsible for cytokine release and extracellular matrix deposition in myelofibrosis. Hyperproliferation and alteration in megakaryocyte morphology are common features in MPNs. (**B**) These aberrant functions are sustained through the hyperactivation of Aurora kinase A (AURKA) and ROCK pathways in megakaryocytes.

Megakaryocytes have an important role in maintaining the hematopoietic stem cell (HSC) quiescence in the bone marrow through cytokine secretion [130]. Megakaryocyte hyperproliferation, impaired differentiation and alteration in their morphology and function are common features in MPNs [131]. ET and PMF patients show abnormal megakaryopoiesis and alteration in their platelet count [132]. Abnormal megakaryocyte differentiation and functionality are considered responsible for cytokine-mediated extracellular matrix deposition in myelofibrosis (Figure 4A). Besides driver mutations inherited by HSC, megakaryocytes may accumulate further mutations not present in other bone marrow cells that may be relevant for their dysregulation and for disease progression [133]. Of note, the inhibition of Aurora kinase A (AURKA) induces differentiation and megakaryocyte polyploidization and provides a therapeutic benefit, reducing disease burden in PMF mouse models [134] and symptom amelioration in human patients [134,135]. The Rho/ROCK pathway is also involved in megakaryocyte differentiation and in platelet production [136,137]. Specifically, a physiological failure to activate the Rho/ROCK pathway during megakaryocyte endomitosis seems to be responsible for polyploidization (Figure 4B) [138]. Furthermore, ROCK overactivation has been identified as a driving force in myeloid proliferation [139,140,141,142,143]. Intriguingly, a JAK2 and ROCK crosstalk has been highlighted in different cell types. JAK2 may activate ROCK through PI3K [139] or by inactivating Rho GAPs [144,145]. On the other hand, ROCK is required for JAK2 downstream signaling by promoting JAK2 phosphorylation, derepressing STAT-mediated transcription or acting as a STAT co-activator [145,146,147,148]. Treatment with ROCK inhibitors have been proved to be effective in preclinical models of acute and chronic myeloid leukemia [139,141,142], which also suggests ROCK as an interesting target in MPNs.

## 4. Drug Combinations: State of the Art

Since MPN patients may lose their response to ruxolitinib because of the dysregulation of the JAK/STAT pathway, with the disease thereby evolving into AML, studies have been conducted to reinforce the therapeutic response. Several combinations of drugs have been tested, including deacetylase and HSP90 inhibitors [149,150], achieving a synergistic efficacy in cell lines and PV and ET mouse models, and overcoming resistance in primary MPN cells by impairing JAK2 stability [151]. In this context, the role of the proviral integration site for the Moloney murine leukemia virus (PIM) kinases [152], ERK1/2 [153], PI3K [154] and mTOR inhibitors [155] has been considered.

Both in cell lines and in primary cells derived from MPN patients, the combination of PIM inhibitors and ruxolitinib synergistically enhanced apoptosis and suppressed colony formation, mediated by BAD and 4EBP1 activation and the inhibition of the mTOR pathway [156] respectively. Recently, Rampal et al. demonstrated that in vitro the triple combination of ruxolitinib, a CDK4/6 inhibitor (LEE011) and a PIM kinase inhibitor (PIM447) resulted in increased levels of apoptosis and a decreased number of cells in the S-phase if compared with ruxolitinib as single agent. In murine models, the combination therapy on one hand contributed to the reduction of liver, spleen and bone marrow fibrosis and on the other hand enhanced the overall survival rate. Moreover, primary cells from MPN patients treated with the same combination showed a lower colony-forming capacity compared with primary cells treated with ruxolitinib only, suggesting that PIM inhibitors together with JAK2 inhibitors could represent a valid therapeutic option [152]. The results of the phase 2 study offering the combination of ruxolitinib and the PI3Kδ inhibitor parsaclisib (INCB050465) in patients with suboptimal response could indicate a potential solution to the problem of persistent PI3K/AKT pathway activation. Interestingly, the phase 3 study will be conducted in treatment-naive patients [157,158].

The PI3K/AKT/mTOR pathway regulates cell growth, cycle progression and metabolism-mediating disease progression by its upregulation. Guglielmelli et al. demonstrated that the mTOR inhibitor everolimus is able to reduce splenomegaly and resolve clinical symptoms [155]. Subsequently, a combination therapy based on PI3K/AKT/mTOR and JAK2 inhibitors has been evaluated in vitro and in mouse models [159], suggesting a new therapeutic option for MF patients. Phase 1 studies with PI3K inhibitors in combination with ruxolitinib (for example NCT02493530) are still ongoing.

There is evidence of the overexpression of HDACs in MPNs, particularly in PMF [160,161]. For these reasons, HDAC inhibitors have been tested in MPN preclinical models and introduced in the clinic. Specifically, given their toxicity and low tolerability, especially in long-term treatments, development strategies are focusing on combinations with JAK inhibitors [162]. HDAC inhibitors such us givinostat and varinostat have been shown to inhibit the proliferation of MPN cells bearing JAK2 V617F. Givinostat inhibits ERK1/2 kinase phosphorylation and also reduces the levels of two transcription factors often up-regulated in MPN patients: NF-E2 and C-MYB [163,164]. Varinostat, on the other hand, is very effective in PV, where it inhibits the activity of the JAK2/STAT5-STAT3 pathways and reduces the phosphorylation of AKT and ERK1/2, thus inhibiting proliferation. In addition, varinostat also reduces NF-E2 levels [165].

JAK2, as described in the paragraph above, phosphorylates the arginine methyltransferase PRMT5. A PRMT5 inhibitor showed relevant efficacy in MPN mouse models and is superior to monotherapy when associated with JAK inhibitors. Mechanistically, PRMT5 inhibition, by deregulating E2F1 methylation, causes alteration in the E2F1 downstream target’s expression, promoting apoptosis [166].

Several studies, both in patients and in preclinical models, demonstrated the key role played by inflammation in the progression of MPNs and in the maintenance of the disease, particularly in the context of MF. These results highlight the relevance of investigating the possible mechanisms underlying this inflammation and an eventual therapeutic combination with JAK inhibitors [118]. NF-κB is an important signaling pathway in the induction of an inflammatory state in MPNs. Indeed, it has recently been shown that by directly binding p65, the BRD4 factor activates its transcriptional activity in vitro and in mouse models of MPNs. In this context, the inhibition of JAK together with BRD4 reduces the inflammatory state and the progression of the disease, delaying the possible emergence of resistance to JAK inhibitors [167]. Uras et al. highlighted that the crucial function of CDK6 in JAK2 V617F-induced MPN progression and maintenance depends on NF-κB regulation. Mechanistically, CDK6 interacts with the p65 subunit, promoting NF-κB transcriptional activity. Moreover, in the context of JAK2 V617F, CDK6 promotes NF-κB signaling by suppressing the transcription of genes that encode its inhibitors [87]. Indeed, the CDK6 inhibitor palbociclib reduces leukocytosis, splenomegaly, and bone marrow fibrosis in mouse models of myelofibrosis, and its efficacy is further potentiated in combination with ruxolitinib. Of note, CDK6 inhibition in hematopoietic stem cells blunts the expression of p65 and Aurora kinases [88].

Numerous drug discovery studies focused on the triggering of the NLRP3 inflammasome through Toll-like receptors (TLRs) and NF-κB activation. The most promising molecules identified for their ability to inhibit this pathway target MyD88 downstream targets, namely IRAK1, IRAK4, and BTK. Among these, TL-895 (a potent irreversible BTK inhibitor) is currently used in phase 2 trials, both alone and in combination with MDM2 inhibitors [168,169] (NCT04655118, NCT04640532).

MPN cell survival and proliferation may depend on the excessive presence of reactive oxygen species (ROS). At the same time, the accumulation of ROS above a certain threshold value can be toxic for cells [170,171]. Nieborowska-Skorska et al. have identified the mechanisms by which ruxolitinib is able to induce an increase in DNA damage and therefore induce an overproduction of ROS. Treatment with ruxolitinib is able to inhibit the two major double-strand-break (DSB) repair mechanisms, BRCA-mediated homologous recombination, and DNA-dependent protein kinase-mediated non-homologous end-joining. This paradoxical mechanism of action of the drug on the one hand is a cause of resistance, and on the other it can be exploited for a possible combinatorial therapy based on the toxicity induced by ROS. Indeed, when combined with the PARP inhibitor olaparib, ruxolitinib caused an abundant accumulation of toxic DSBs, resulting in an overproduction of ROS and enhancing the elimination of MPN primary cells [172].

Given the important role of the MAPK pathway in reducing the efficacy of JAK inhibitors, the targeting of this signaling could represent a beneficial therapeutic approach. MAPK pathway inhibitors such as BRAF and MEK are already in use in MPNs. In particular, several therapeutic benefits have been observed with the MEK inhibitors binimetinib, selumetinib, trametinib and PD0325901 when combined with JAK2 inhibition [81,173]. Very recently, Sime Brkic et al. demonstrated that the genetic and pharmacological targeting of ERK1/2 reduces the characteristic features of MPNs. Furthermore, the combined inhibition of JAK2 and ERK1/2 with ruxolitinib and ERK inhibitors, respectively, reduces the proliferation of Jak2 V617F cells, normalizes erythrocytosis and corrects the splenomegaly of Jak2 V617F MPN mice [153].

Finally, among the various possible candidates for a combinatorial therapy with ruxolitinib there are also p53 activators and pegylated interferon alfa-2a (PEG-IFNa2). MDM2 is overexpressed in MPN progenitor cells carrying the JAK2 V617F mutation, promoting p53 degradation. MDM2 inhibitors, such as RG7112, have been shown to reduce MPN colony formation by mediating the preferential eradication of JAK2 V617F-expressing progenitors. Of note, MDM2 inhibitors are currently in clinical trials in patients with PV or ET [174,175]. Promising data on the use of the Navtemadlin MDM2 inhibitor (KRT-232), including potential disease-modifying activities, have recently been announced [176]. PEG-IFNa2 has been demonstrated to improve MPN-related symptoms, obtaining a superior clinical response in PV and ET patients intolerant to HU [177]. PEG-IFNa2 induced durable hematological and molecular responses in older patients with early-stage PV, demonstrating its non-inferiority to HU treatment [178]. Its efficacy and safety were also tested in combination with ruxolitinib and a remission rate of 31% for PV patients and 44% for MF patients was observed, resulting in both JAK2 V617F allele burden decreasing and a reduction in inflammation [45].

## 5. Multidrug Resistance: Lesson from Other Cancers

The concept of multidrug resistance (MDR) is a subject of constant debate, not only with respect to other hematological diseases, such as multiple myeloma (MM) and AML, but also in solid tumors. In MM, combination therapy has made significant progress, up to the proposal of quadruplet regimens composed of proteasome inhibitors, anti-CD38 antibodies, immunomodulatory drugs and steroids. However, outside the core of the treatment strategy, there are known escape mechanisms, often caused by secondary genetic events of the cytogenetic type (mainly 17p and 13p, underlying alterations associated with c-Myc) or involving target anti-apoptotic mutations (e.g., TP53, NRAS, KRAS, and BRAF) [179]. Interestingly, the same mutations predict resistance to anti-EGFR immunotherapy in patients with metastatic colorectal cancer [180]. Among the pharmacological novelties to reactivate tumor suppressors, histone deacetylase inhibitors such as panabinostat and vorinostat, both capable of opening the chromatin structure, should be mentioned. Selinexor, which by inhibiting esportin 1 (XP01) contributes to blocking the export tumor suppressor proteins inside the cytoplasm and to the maintenance of oncoprotein mRNAs inside the nucleus, could have an important role in combination strategies [181].

AML has a genetically complex terrain and MDR has been and still is the subject of numerous studies. It is not the aim of this manuscript to go into the details of this disease; however, where hypomethylating agents and venetoclax had partly answered the dilemma of chemoresistance, the cases of refractory to the most recent combinations are an excellent example of triggering pathways alternative to those inhibited. With venetoclax, the contributions of Bcl-XL (inhibited by navitoclax), or of the induced myeloid leukemia cell differentiation protein (Mcl-1), as well as the loss of the pro-apoptotic protein BAX are frequent possibilities [182]. Likewise, drug efflux via P-gp renders the CD33^+^ cell resistant to gentuzumab ozagamicin. Hence, the research of MDR modifiers becomes crucial for optimizing association therapies [183].

## 6. Conclusions

The comprehension of aberrant signal transduction and of the mechanisms of adaptation to targeted therapies occurring in MPN cells is of extreme importance to define successful combinatorial treatments. As in other neoplastic cells, signal transduction pathways inhibited by drug treatment may resume thanks to the specific activity of inhibitors or to anomalous cross-activation among pathways. A clear picture of these atypical connections may help in drawing a map to deploy a crossfire on the right targets. This goal is made challenging by the difficulty of studying HSCs in their niche and general hematopoietic cells in their specific contexts, which respond to different types of environmental stimuli. A possibility is to evaluate the signaling pathway activation directly on bone marrow biopsies. The availability of antibodies recognizing the phosphorylated forms of signaling molecules suitable for immunohistochemical analysis may allow a more precise evaluation of signal transduction alteration in patients. Mass cytometry analysis may also represent an important tool to dissect at the single-cell level signaling pathway alterations and to highlight differences in the expression and activation of signaling molecules during treatments and progression. All these efforts will likely add new important information to fight malignant clone persistence in MPNs.

The overcoming of the model based solely on the containment of cell proliferation has, however, produced important effects on translational research, and on the introduction of interesting combination therapies. Myelofibrosis remains a pathology of enormous biological complexity, and we can no longer exempt ourselves from studying this complexity in every patient. The future will thus be made up of tailored therapeutic strategies, adapted to the acquisition of resistance phenomena. In addition, all drugs that appear to be capable of modifying disease burden and fibrosis deserve special attention. MDR represents a broad topic of study, which arises at different times and contexts, depending on the disease treated. Going down into the facts, we can see several similarities between MPNs, AML, MM and solid cancers. Some diseases have a consolidated experience of target therapies and pharmacological associations are already available. Comparative studies on the ignition of alternative pathways could lead to the discovery of MDR modifiers to be used across the board.

## Figures and Tables

**Table 1:** Some clinical trials including JAK2 inhibitors for MPN patients. PPV-MF, post-polycythemia vera myelofibrosis; PET-MF, post-essential thrombocythemia-myelofibrosis.

Clinical Trial	Type of Inhibitor	Setting of Disease	Reference
COMFORT-I	Ruxolitinib, (JAK1/2 inhibitor)	MF, PPV-MF, PET-MF	[36]
COMFORT-II	Ruxolitinib, (JAK1/2 inhibitor)	Intermediate-2 or high-risk MF, PPV-MF, PET-MF	[37]
SIMPLIFY-1	Momelotinib, (JAK1/2 and ACVR1 inhibitor)	High-risk, intermediate-2-risk, or symptomatic intermediate-1-risk-naive MF	[38]
SIMPLIFY-II	Momelotinib, (JAK1/2 and ACVR1 inhibitor)	MF with suboptimal responses or haematological toxic effects with ruxolitinib	[39]
RESPONSE	Ruxolitinib, (JAK1/2 inhibitor)	Jak-inhibitor-naive PV	[40]
PERSIST-1	Pacritinib, (JAK2, IRAK1 and FLT3 inhibitor)	High-risk MF	[41]
PERSIST-2	Pacritinib, (JAK2, IRAK1 and FLT3 inhibitor)	Intermediate-1, intermediate-2, or high-risk primary or secondary MF	[42]
JAKARTA-1	Fedratinib, (JAK2, RET and FLT3 inhibitor)	Primary or secondary MF	[43]
JAKARTA-2	Fedratinib, (JAK2, RET and FLT3 inhibitor)	Intermediate- or high-risk MF, PPV-MF, or PET-MF previously treated with Ruxolitinib	[44]
COMBI	Ruxolitinib, (JAK1/2 inhibitor) and Interferon-α2	MF and PV	[45]
PACIFICA	Pacritinib, (JAK2, IRAK1 and FLT3 inhibitor)	MF, PPV-MF, PET-MF	[46]

**Table 2:** Some of ongoing clinical trials with novel agents other than JAK2 inhibitors. PPV-MF, post-polycythemia vera myelofibrosis; PET-MF, post-essential thrombocythemia-myelofibrosis.

Agent	Disease Setting	Clinical Trial	Phase
Elotuzumab (anti CD319)	MF	NCT04517851	Phase 2
Selinexor (SINE inhibitor)	Naive MF	NCT04562389	Phase 1/2
CPI-0610 (BET inhibitor)	MF, PPV-MF, PET-MF	NCT04603495	Phase 3
Imetelstat (Telomerase inhibitor)	Intermediate-2- or high-risk MF refractory to JAK inhibitor	NCT04576156	Phase 3
Alisertib (AURKA inhibitor)	PMF	NCT02530619	Pilot study
Navitoclax (Bcl-2 inhibitor)	MF/Relapsed/Refractory MF	NCT04454658/NCT04468984	Phase 1/Phase 3
TL-895 (BTK inhibitor)	MF	NCT04655118	Phase 2
Navtemadlin (MDM2 inhibitor)	MF, PPV-MF, PET-MF with suboptimal response to Ruxolitinib	NCT04485260	Phase 1b/2
Navtemadlin (MDM2 inhibitor)	MF, PPV-MF, PET-MF	NCT03662126	Phase 2/3
Navtemadlin (MDM2 inhibitor) + TL-895 (BTK inhibitor)	MF, PPV-MF, PET-MF	NCT04640532	Phase 1/2
Ruxolitinib (JAK1/2 inhibitor) + Parsaclisib (PI3Kδ ihibitor)	MF, PPV-MF, PET-MF	NCT04551066	Phase 3

**Table 3:** Direct and indirect JAK2 downstream targets involved in JAK2 inhibitor resistance and disease persistence.

JAK2 Downstream Targets	Function	Localization	Mechanism of Action
STATs	Signal transduction and activation of transcription	Cytoplasm and nucleus	STAT target gene transcription
PI3K/AKT/mTOR	Signal transduction	Cytoplasm	Increased cell survival and proliferation and regulation of cell metabolism [79]
ERK1/2	Signal transduction	Cytoplasm	Cell survival and proliferation [69]
Histone H3	Chromatin folding and accessibility	Nucleus	Chromatin decondensation and increased gene expression (i.e., *lmo2*) [66]
KDM3A	Histone demethylase	Nucleus	Enhanced STAT3 target gene transcription [69]
PRMT5	Histone methyltransferase	Nucleus	Inhibition of PRMT5 methyltransferase function, gene transcription alteration [79]
YBX1	Splicing factor	Cytoplasm and nucleus	Sustained ERK signaling and disease persistence [69]
PIM	Signal transduction	Cytoplasm and nucleus	Cell survival, proliferation, metabolism, and drug resistance [69,85]
MDM2	Ubiquitin ligase	Cytoplasm	p53 degradation, increased cell survival, and proliferation [86]
CDK6	Cyclin dependent kinases	Nucleus	Sustained NF-kB signaling, cytokine secretion [87,88]
BTK	Signal transduction	Cytoplasm and nucleus	Cell migration [89]
NLRP3 inflammasome	Cleavage of the precursors form of IL-1β and IL-18	Cytoplasm	Maturation and secretion of pro-inflammatory IL-1β and IL-18 [69]
